# Supplementary material for: Development of a computer-aided design software for the quantitative evaluation of aesthetic damage
Source: PLoS One. 2019 Dec 18;14(12):e0226322. doi: 10.1371/journal.pone.0226322 (PMC6919621; doi:10.1371/journal.pone.0226322)
Supplement: S1 Code site — 1. (ZIP) [file pone.0226322.s001.zip › [EESC_jr][Projeto]Relatorio_07_21/reports/templates_zip/gentelella-master/vendors/jQuery-Smart-Wizard/more_examples/smartwizard2-validation.php]

Smart Wizard 2 - Step Validation Example - a javascript jQuery wizard control plugin

## Step Validation Example

if(isset($\_REQUEST['issubmit'])){
echo "<strongform is sumbitted";
}
?>

- 1

  Account Details  
  Fill your account details
- 2

  Profile Details  
  Fill your profile details
- 3

  Contact Details  
  Fill your contact details
- 3

  Other Details  
  Fill your other details

## Step 1: Account Details

|  |  |  |
| --- | --- | --- |
|  | | |
| Username : |  |  |
| Password : |  |  |
| Confirm Password : |  |  |

## Step 2: Profile Details

|  |  |  |
| --- | --- | --- |
|  | | |
| First Name : |  |  |
| Last Name : |  |  |
| Gender : | -select- Female Male |  |

## Step 3: Contact Details

|  |  |  |
| --- | --- | --- |
|  | | |
| Email : |  |  |
| Phone : |  |  |
| Address : |  |  |

## Step 4: Other Details

|  |  |  |
| --- | --- | --- |
|  | | |
| Hobbies : |  |  |
| About You : |  |  |
